# Palivizumab in the prevention of severe respiratory syncytial virus infection in children with congenital heart disease; a novel cost-utility modeling study reflecting evidence-based clinical pathways in Spain

**DOI:** 10.1186/s13561-017-0181-3

**Published:** 2017-12-19

**Authors:** Ralph Schmidt, Istvan Majer, Natalia García Román, Alejandra Rivas Basterra, ElizaBeth Grubb, Constancio Medrano López

**Affiliations:** 1 Pharmerit International, Health Economics and Outcomes Research, Zimmerstraße 55, 10117 Berlin, Germany; 2 Pharmerit International, Health Economics and Outcomes Research, Rotterdam, the Netherlands; 3 AbbVie, Medical Department, Madrid, Spain; 4 AbbVie, Government Affairs and Market Access, Madrid, Spain; 5 AbbVie, Health Economics and Outcomes Research, Chicago, IL USA; 6 Pediatric Cardiology, Gregorio Marañón University Hospital, Madrid, Spain

**Keywords:** Palivizumab, Prophylaxis, Cost-effectiveness, Respiratory syncytial virus, Congenital heart disease, Spain

## Abstract

**Background:**

Respiratory syncytial virus (RSV) infection remains one of the major reasons of re-hospitalization among children with congenital heart disease (CHD). This study estimated the cost-effectiveness of palivizumab prophylaxis versus placebo, in Spain, from the societal perspective, using a novel cost-effectiveness model reflecting evidence-based clinical pathways.

**Methods:**

A decision-analytic model, combining a decision tree structure in the first year and a Markov structure in later years, was constructed to evaluate the benefits and costs associated with palivizumab versus no prophylaxis among children with CHD. In the first year of the model, children were at risk of mild (i.e. medically attended, MA-RSV) and severe (hospitalized, RSV-H) RSV infection. The impact of delayed corrective CHD surgery due to RSV infection and the consequence of performed surgery despite severe infection were considered. In later years, patients were at risk of developing asthma and allergic sensitization as sequelae of RSV infection. Input data for the model were derived from the pivotal clinical trial and systematic literature reviews. Indirect costs included parental absence from work and nosocomial infections. In agreement with Spanish guidelines, costs and effects were discounted at 3%.

**Results:**

Over a lifetime horizon, palivizumab prophylaxis yielded 0.11 and 0.07 additional quality-adjusted life years (QALYs) and life years (LYs), respectively, at additional costs of € 1,693, resulting in an ICER of € 15,748 per QALY gained and € 24,936 per LY gained. Probabilistic sensitivity analyses demonstrated that the probability of palivizumab prophylaxis being cost-effective at a € 30,000 per QALY threshold was 92.7%. The ICER remained below this threshold for most extreme scenario analyses.

**Conclusions:**

The model demonstrated that palivizumab prophylaxis results in more QALYs than no prophylaxis in children with CHD. Palivizumab prophylaxis was shown to be a cost-effective health care intervention according to the commonly accepted standards of cost-effectiveness in Spain (ICER below the threshold of € 30,000 per QALY).

## Background

The human respiratory syncytial virus (RSV) is known to cause acute lower respiratory tract infections (LRTIs) during infancy and childhood. RSV activity can vary geographically, with the peak season for temperate climates lasting any time between November and April [1, 2]. Approximately, 60% of infants are infected during their first RSV season and almost all by the age of two [3]. In these infants, RSV infection may result in complications (e.g. bronchiolitis and acute respiratory or ventilatory failure) necessitating hospitalization and sometimes mechanical ventilation with transfer to an intensive care unit (ICU) [4]. Globally, 34 million episodes of acute LRTIs, 3.4 million hospitalizations and 199,000 deaths are estimated to occur due to RSV each year [3].

The clinical burden associated with RSV infection differs considerably between patients. In most cases, RSV infection presents itself as a common cold and requires no medical attention. However, in other cases, patients have serious negative health consequences that are known to result in respiratory problems that can last into adulthood. The economic burden of RSV has been estimated at an annual cost of € 47 million for the Spanish National Health Care System [5].

In general, three subgroups of children are considered to be at increased risk of severe infection: children with congenital heart disease (CHD), children with chronic lung disease (CLD), and infants who are prematurely born (i.e. born prior to the 37th week of gestation). Within this latter group, children are further stratified by their gestational age and the presence of additional risk factors such as contact with other children and lack of breast feeding [6]. In the present cost-utility analysis, we focus on children with CHD.

Given the significant humanistic and economic burden of RSV infection in high risk groups [5–8], prevention of infection is highly recommended by the Spanish Association of Pediatrics (*Asociación Española de Pediatría*) [9, 10] as well the Spanish Society of Pediatric Cardiology and Congenital Heart Disease (*Sociedad Española de Cerdiolohia Pediatrica y Cardiopatias Congenitas*) [11]. To date, no RSV vaccine or antiviral therapy exists and the most effective method of prevention is prophylaxis with palivizumab [12]. Palivizumab is an FDA- and EMA-approved prescription injection of RSV-targeting antibodies and was granted access to most markets in the late 1990s [2]. During an RSV season, it is administered monthly via the intramuscular route, at a dose of 15 mg/kg [13].

The efficacy of palivizumab has been demonstrated in three randomized, double-blind, placebo-controlled clinical trials: the IMpact trial (*n* = 1502), [14] the MAKI trial (*n* = 429), [15] and the Feltes trial (*n* = 1287) [16]. The IMpact trial investigated the reduction in RSV-related hospitalizations among premature infants or children with CLD during the first RSV season of their life. Results from this trial showed that palivizumab prophylaxis was associated with a 55% (95% confidence interval [CI] 38–72%) reduction in hospitalizations. The more recent MAKI trial focused on parent-reported wheezing patterns in late preterm infants who received palivizumab or placebo (no prophylaxis). The study demonstrated a 47% (95% CI 14–80%) reduction in the number of patients suffering from recurrent wheezing and proved that palivizumab treatment significantly reduced the need for RSV-related outpatient visits in mild cases of RSV infection. The Feltes study included children (≤2 years of age) with CHD and demonstrated that palivizumab recipients had a 45% (95% CI 23–67%) relative risk reduction (RRR) in RSV hospitalizations.

Previously, several health economic evaluations comparing palivizumab prophylaxis with placebo have been performed [17–25]. Among these, a handful of evaluations focused on children with CHD [24–27]. Although the evaluations differed in terms of the modeling approach, input data, model outcomes and the estimated results, none of the studies sufficiently captured the impact of the complicated disease course in children with CHD. Specifically, the risk of delayed and complicated heart surgery as a severe RSV-related complication was not incorporated. Additionally, previously published studies did not consider patients with MA-RSV, long-term respiratory sequelae of RSV infection (asthma and allergic sensitization), and the impact of hospital-acquired nosocomial infections. The present study assessed the cost-utility of prophylaxis with palivizumab versus no prophylaxis in children with CHD in Spain, using a health economic model that captured the complexities of the RSV disease course as well as the specific clinical pathway of CHD patients. The analysis was conducted from a societal perspective.

## Methods

### Model structure

**Fig. 1:**
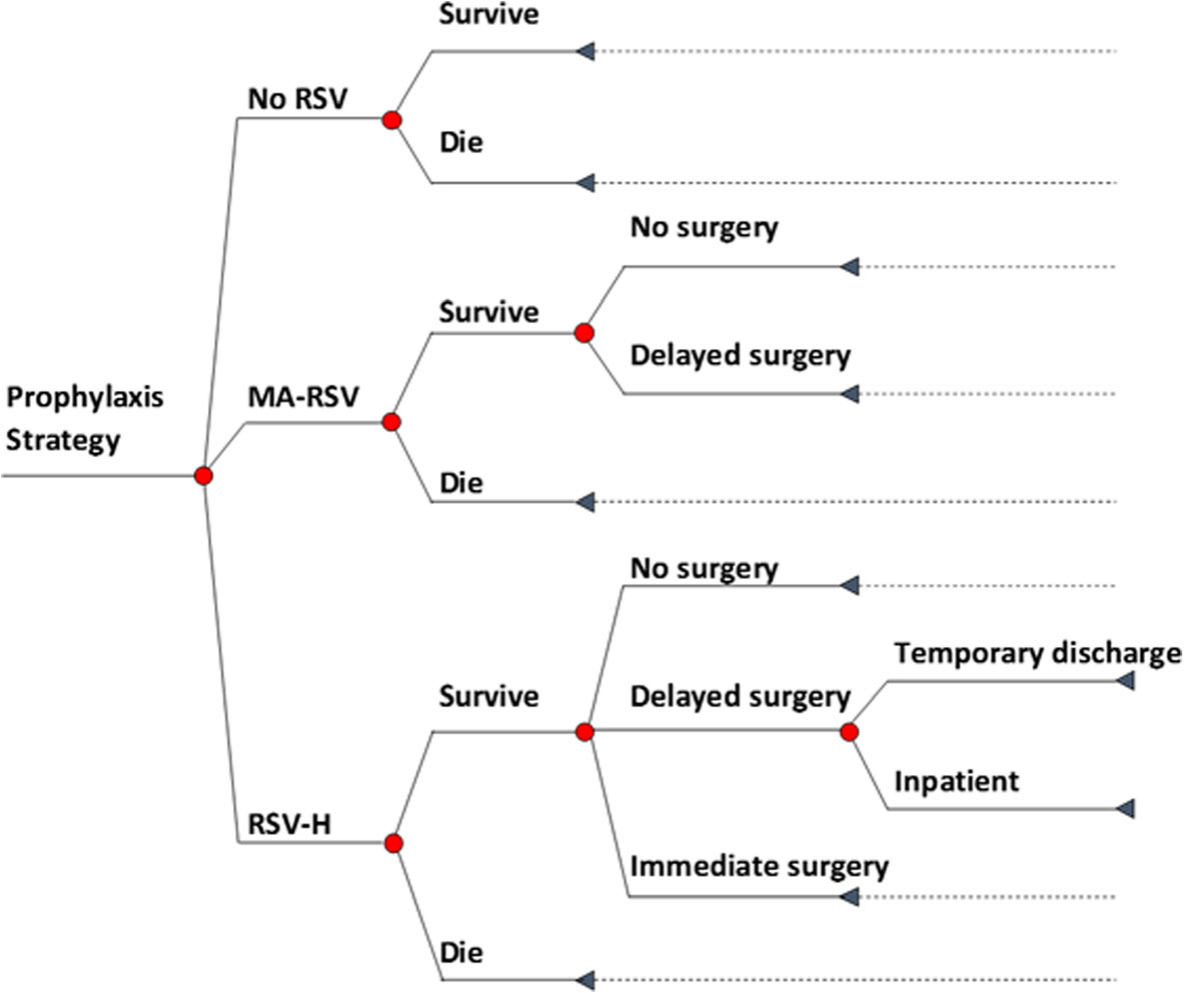
Decision tree model structure for children with CHD during the first year of the model simulation. Abbreviations: RSV, respiratory syncytial virus; MA-RSV, medically attended RSV infection; RSV-H, RSV infection resulting in hospitalization

**Fig. 2:**
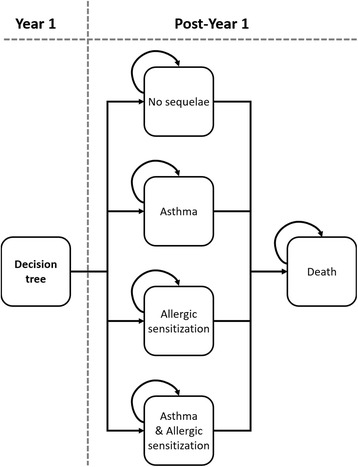
Markov model structure for children with CHD after the first year of the model

A decision-analytic model was developed in Microsoft Excel 2016, combining a decision tree structure for the first year (see Fig. 1) and a Markov structure for subsequent years (annual cycles, please see Fig. 2). In line with the Feltes trial design, patients received either palivizumab or placebo (i.e. no prophylaxis) during their first RSV season (year 1).

Since most children with CHD require cardiac surgery to correct the heart defects early in life, the decision tree structure applied in the first year was deemed appropriate to capture potential complications in children scheduled for heart surgeries. Children could experience three possible events during this first year: no identified RSV infection (‘No RSV’ branch), medically attended RSV infection (‘MA-RSV’) or RSV hospitalization (‘RSV-H’). Among those who were hospitalized for severe RSV infection, three possible events were further considered: ‘No (planned) surgery’ (due to either not requiring surgery at all or having had corrective surgery before the infection), ‘Delayed surgery’ (i.e. surgery postponed until after RSV infection is cleared), and ‘Immediate surgery’, with consequences of increased health care resource utilization. Regarding MA-RSV patients, immediate surgery was deemed not to be a clinically relevant outcome, because these patients, by definition, were not in the hospital at the time of the RSV infection and any scheduled surgery must have been further away in time. Therefore, RSV-related complications associated with immediate surgery did not need to be captured for the MA-RSV patients in the model.

After the first year of the simulation, patients entered the Markov model where their health status was evaluated annually (i.e. every model cycle) over a lifetime horizon (i.e. until the age of 100 years). It has been established that the health impact of RSV infection extends beyond the acute episode phase [28]. In particular, significant risk increases in developing asthma and allergy after RSV infection in the first year of life have been documented, even at the age of 18 years [29]. Therefore, the health states considered in the Markov model were directly related to the presence of long-term respiratory sequelae. Four mutually exclusive health states were distinguished: asthma, allergic sensitization, both asthma and allergic sensitization, and no long-term sequelae. Patients remained in these health states until 18 years of age or death. The modeling approach was validated by a pediatric cardiologist who is an expert in the disease field.

### Efficacy input parameters

All efficacy input parameters are presented in Table 1, whereas the transition probabilities from the decision tree to the Markov model and related calculations are shown in Table 2. The risks of RSV-H and MA-RSV were based on the results of the pivotal trial of Feltes et al. [16] and the MAKI trial [15], respectively. The risks of immediate and delayed surgery were derived from a United States (US)-based retrospective study, that assessed hospital charts of children with a pre-RSV infection cardiac diagnosis [30]. Patients in this retrospective study were categorized as either surgical or medical. Surgical patients were children who underwent congenital heart surgery and had RSV infection during the same hospitalization. Medical patients were children with CHD who were hospitalized with RSV infection but did not undergo cardiac surgery during the admission for RSV. Medical patients were distinguished by delayed surgery and no surgery. In addition to this, patients with delayed surgery were further categorized into inpatient (i.e. requiring hospitalization until the surgery) and outpatient (i.e. discharged to home until the surgery) subgroups. The probabilities of patients arriving at each of the modeled branches were taken from this retrospective study [30], except for the inpatient and outpatient subgroups within patients with delayed surgery, for which input data was informed by clinical expert opinion.

**Table 1:** Efficacy input parameters

Parameter	Palivizumab (SE)	No prophylaxis (SE)	Distribution in PSA	Source
First year (decision tree)				
MA-RSV risk, %	1.9 (0.4)	8.1 (1.6)	Beta	MAKI trial (2013, Feltes et al. (2003)
RSV-H risk, %	5.3 (0.9)	9.7 (1.9)	Beta	Feltes et al. (2003)
RSV-H: Immediate surgery, %	11.1 (4.0)	11.1 (4.0)	Beta	Altman et al. (2000)
RSV-H: No immediate surgery, %	88.9 (17.8)	88.9 (17.8)	Beta	Altman et al. (2000)
No surgery, %	39.3 (7.9)	39.3 (7.9)	Beta	Altman et al. (2000)
Delayed surgery, %	60.7 (6.5)	60.7 (6.5)	Beta	Altman et al. (2000)
Inpatient vs discharged before delayed surgery, %	33 vs 67 (8.1)	33 vs 67 (8.1)	Beta	Expert opinion*
Case fatality, %	5.2 (0.9)	5.2 (0.9)	Beta	Szabo et al. (2013)
CHD-specific background mortality (first 20 years)	age-specific mortality	age-specific mortality	Fixed	Tennant et al. (2010)
CHD-specific background mortality (beyond 20 years)	age-specific mortality	age-specific mortality	Fixed	Diller et al. (2015), National mortality statistics (Spain, 2012)
Proportions of respiratory sequelae (used for transition probability calculations, see Table 2)				
*MA-RSV:*				
Asthma, %	10.3 (1.1)	10.3 (1.1)	Beta	Stein et al. (1999)
Allergic sensitization, %	37.4 (3.9)	37.4 (3.9)	Beta	Sigurs et al. (2010)
*RSV-H:*				
Asthma, %	32.6 (6.9)	32.6 (6.9)	Beta	Stein et al. (1999)
Allergic sensitization, %	43.5 (7.3)	43.5 (7.3)	Beta	Sigurs et al. (2010)
Other parameter				
Nosocomial infection, %	6.1 (0.6)	6.1 (0.6)	Beta	Ehlken et al. (2005)

*The SE was assumed to be 20% of the estimated mean

Abbreviations: SE, standard error; PSA, probabilistic sensitivity analysis; RSV, respiratory syncytial virus; MA-RSV, medically attended RSV infection; RSV-H, RSV infection resulting in hospitalization

**Table 2:** Transition probabilities from the decision tree to the Markov model

Parameter	Transition probability (Palivizumab and no prophylaxis)	Calculation (see Table 1 for efficacy inputs)
MA-RSV		
No sequelae, %	56.1	(1 − *p*_*Asthma*_) × (1 − *p*_*AS*_)
Asthma, %	6.4	*p*_*Asthma*_ × (1 − *p*_*AS*_)
Allergic sensitization, %	33.6	(1 − *p*_*Asthma*_ ) × *p*_*AS*_
Asthma and allergic sensitization, %	3.9	*p*_*Asthma*_ × *p*_*AS*_
RSV-H		
No sequelae, %	38.1	(1 − *p*_*Asthma*_) × (1 − *p*_*AS*_)
Asthma, %	18.4	*p*_*Asthma*_ × (1 − *p*_*AS*_)
Allergic sensitization, %	29.3	(1 − *p*_*Asthma*_ ) × *p*_*AS*_
Asthma and allergic sensitization, %	14.2	*p*_*Asthma*_ × *p*_*AS*_

Abbreviations: RSV, respiratory syncytial virus; MA-RSV, medically attended RSV infection; AS, allergic sensitization; RSV-H, RSV infection resulting in hospitalization

The model made a distinction between two types of mortality, one that reflected the elevated mortality among children with CHD hospitalized for RSV, referred to as case fatality, and one that related to any other cause of death, referred to as background mortality. The case fatality rate was applied only in the first year of the simulation (i.e. in the decision tree) and was applied to RSV-H patients only, whereas the background mortality was applied annually throughout the whole simulation to all patients. The case fatality risk was applied equally to both model arms, assuming no effect of prophylaxis on the risk of death for RSV-H children. It was derived from a meta-analysis study that included several articles reporting case fatality rates in young children with CHD hospitalized for severe RSV LRTI [31], and has been used in a previous modeling study [24]. The CHD-specific background mortality was sourced from two publications and was applied to all years of the simulation. For the first 20 years of the simulation, the model made use of mortality data published by Tennant et al. [32], who reported a 1-year survival of 92.3% and a 20-year survival of 89.5% in children with different types of congenital anomalies of the cardiovascular system. Based on these figures, the background mortality risk applied in the model was 7.7% for the first year and 0.16% for years 2–20 (risk of death over a 19-year period, i.e. 1–89.5%/92.3%, annualized). Beyond 20 years, the model was informed by age-specific general population mortality data from the national mortality statistics published for Spain (2012) [33] that was adjusted by the difference between the adult CHD population and the general population (standardized mortality ratio [SMR]: 2.29 as published in Diller et al. [34]).

MA-RSV and RSV-H have been identified as possible risk factors for asthma (or recurrent wheezing) and allergic sensitization in genetically predisposed children [29, 35, 36]. The distribution over the Markov health states, at the moment of transitioning from the first year (decision tree) to the second year, was informed by two studies, Sigurs et al. [29] and Stein et al. [36], that reported estimates of the association of RSV infection (severe and mild) and long-term respiratory sequelae. We used different incidence rates of respiratory sequelae for patients with MA-RSV and RSV-H. Patients without an MA-RSV or RSV-H infection were assumed to be free of RSV-induced respiratory illnesses. Probabilities of sequelae, along with their respective calculations, are outlined in Table 2. Based on the findings of the Sigurs et al. study [29], respiratory sequelae were assumed to last 18 years.

A novel aspect of the current model is the inclusion of nosocomial infections. The transmission risk, i.e., the risk that children with RSV-H can infect other hospitalized children was taken from a publication by Ehlken et al. which investigated the impact of nosocomial-acquired LRTIs in young children [37]. In the model, we assumed that whenever a nosocomial infection occurs, only one RSV-H patient transmits the infection to only one other child and the cost of such nosocomial infections were considered.

### Utilities

Utility values for the different health states were derived from published literature and are presented in Table 3. Greenough et al. conducted a retrospective study on health-related quality of life (HRQoL) in prematurely born children in the United Kingdom (UK) [38]. The study compared the HRQoL of children with RSV-H and without RSV-H at the age of five years, using the Health Utilities Index (HUI) measure. The median HUI was 0.88 and 0.95 in the two groups of children, respectively. The mean scores were not reported. For the purpose of the model, it was assumed that the decrement of 0.070 was applicable for the first five model cycles (decision tree and four Markov cycles). The same utility values were used in previous cost-utility models [17, 21, 24, 39–41]. After the age of five years, HRQoL was assumed to be impacted only by the presence of RSV-induced respiratory sequelae. The disutility for asthma (0.048) was obtained from a publication by Briggs et al. [42], who investigated the cost-effectiveness of asthma control in the UK, by using data from the Gaining Optimal Asthma Control (GOAL) study. The disutility of allergic sensitization (0.046) was reported in a publication by Brüggenjürgen et al. [43], who evaluated the cost-effectiveness of specific subcutaneous immunotherapy in addition to symptomatic treatment compared with symptomatic treatment alone, in a German health care setting. These decrements in utility due to sequelae were assumed independent and (in the absence of further evidence) additive. Thus, a child who suffered from both asthma and allergic sensitization had an HRQoL loss of 0.094. Furthermore, in line with other publications [24, 40, 41], the baseline utility value of 0.95 was assumed to be applicable until patients reach adulthood (18 years), after which there was no modeled RSV-associated impact on utility (sequelae assumed to last 18 years). From 18 years onwards, the simulated cohort had Spanish-specific utilities, which were sourced from Szende et al. [44]. These utility values were 0.982, 0.975, 0.949, 0.923, 0.901, 0.891, and 0.781 for the age groups 18–24 years, 25–34 years, 35–44 years, 45–54 years, 55–64 years, 65–74 years, and 75+ years, respectively.

**Table 3:** Utility and cost parameters

	Palivizumab/placebo (SE)	Distribution in PSA	Source
Utilities			
Baseline (age 0–17 years)	0.950 (0.162)	Beta	Greenough et al. (2004)
Baseline (age 18–24 years)	0.982 (0.003)	Beta	Szende et al. (2014)
Baseline (age 25–34 years)	0.975 (0.003)	Beta	Szende et al. (2014)
Baseline (age 35–44 years)	0.949 (0.009)	Beta	Szende et al. (2014)
Baseline (age 45–54 years)	0.923 (0.010)	Beta	Szende et al. (2014)
Baseline (age 55–64 years)	0.901 (0.009)	Beta	Szende et al. (2014)
Baseline (age 65–74 years)	0.891 (0.007)	Beta	Szende et al. (2014)
Baseline (age 75 years onwards)	0.781 (0.014)	Beta	Szende et al. (2014)
Decrement: RSV-H	0.070 (0.014)	Beta	Greenough et al.(2004)
Decrement: asthma	0.048 (0.010)	Lognormal	Briggs et al. (2006)
Decrement: allergic sensitization	0.046 (0.009)	Lognormal	Brüggenjürgen et al. (2008)
Direct costs			
Prophylaxis cost	€ 2902	Fixed	Synagis vial cost, Pedraz et al. (2003), Clinical expert input
General ward hospital stay/day	€ 591 (€ 118)	Gamma	Lázaro y de Mercado et al. (2006)
Intensive care support/day	€ 1041 (€ 208)	Gamma	Lázaro y de Mercado et al. (2006)
Outpatient visit	€ 21 (€ 4)	Gamma	Dal Negro et al. (2007)
Asthma/year	€ 744 (€ 149)	Gamma	Blasco Bravo et al. (2011)
Allergy/year	€ 198 (€ 40)	Gamma	Smith et al. (2005)
RSV-H (pediatric ward), days	7 (1.4)	Gamma	Medrano et al. (2010)
RSV-H – ICU, days	10 (2.0)	Gamma	Medrano et al. (2010)
Risk of ICU admission, %	30.4 (6.1)	Beta	Medrano et al. (2010)
Delayed surgery (outpatient), GP visits	4 (0.8)	Gamma	Expert opinion
Delayed surgery (inpatient), days	28 (5.6)	Gamma	Expert opinion
Immediate surgery, days	2.1 (0.42) length of stay in pediatric ward	Gamma	Altman et al. (2000)
Indirect costs			
Missed work: Palivizumab administration, hours	2 (0.4)	Gamma	Assumption
Missed work: MA-RSV, hours	2 (0.4)	Gamma	Assumption
Missed work: RSV-H, hours	57 (11.5)	Gamma	Medrano et al. (2010), Assumption
Asthma/year	€ 495 (€ 99)	Gamma	Blasco Bravo et al. (2011)
Allergy/year	€ 259 (€ 52)	Gamma	Smith et al. (2005)
Nosocomial infection: risk, %	6.1 (0.6)	Beta	Ehlken et al. (2005)
Nosocomial infection (pediatric ward), days	14 (2.8)	Gamma	Assumption, Expert opinion
Absence from work/hour	€ 20 (€ 4)	Gamma	*Anuario Estadístico de Espana* (2017; Spanish statistical yearbook)

Notes: Presented costs were either 2016 costs or were inflated to 2016 costs

Abbreviations: GP, general practitioner, SE, standard error; PSA, probabilistic sensitivity analysis; RSV, respiratory syncytial virus; RSV-H, RSV infection resulting in hospitalization; ICU, intensive care unit; MA-RSV, medically attended RSV infection

### Costs

All cost inputs used in the model are presented in Table 3. In line with Spanish guidelines, the analysis considered the societal perspective meaning that both direct and indirect costs were accounted for in the model. Cost data for the economic model were obtained from Spanish national databases and published literature (see Table 3 for references). Furthermore, all costs reported are in 2016 euros (€).

Palivizumab drug costs were estimated based on the average 50 mg (€ 434.91) and 100 mg (€ 722.19) vial consumptions across all administrations. In line with the label of palivizumab, no vial sharing was taken into account. Using local clinical expert input, it was assumed that on average 5% of the administrations required a 50 mg vial and 95% of the administrations required a 100 mg vial. To obtain the total cost of prophylaxis, the per-protocol palivizumab costs (five administrations for all children) were adjusted for the actual number of administrations (4.1 injections on average), as reported in Pedraz et al. [45] for Spain.

Hospitalization costs for the first year were calculated by multiplying the daily costs of stay in a pediatric general ward and/or a pediatric ICU by the corresponding numbers of days a patient spent in these wards. It was assumed that all hospitalized patients were admitted to the general ward, whereas only a small group of children were admitted to the ICU. Length of stay in the general ward and ICU, as well as the risk of ICU admission were derived from the recent Spanish CIVIC study [46]. The daily costs of a stay in pediatric general ward and pediatric ICU were derived from Lázaro y de Mercado et al. [19]. It was assumed that the cost associated with MA-RSV was a single GP visit. The cost of a GP visit was derived from Dal Negro et al. [47].

Indirect costs in the first year included lost productivity costs of parents associated with palivizumab administration, for MA-RSV and/or RSV-H children. The cost of lost productivity associated with RSV-H was calculated based on the length of hospital stay. The average wage in Spain (2016 wage) [48], eight working hours per day, and five working days per week were considered.

The cost associated with a nosocomial infection was treated as an indirect cost because nosocomial infections did not directly impact the modeled cohort, i.e. the newly infected, hospitalized children were not part of the initial RSV-H cohort. Based on expert input, cost of two weeks of inpatient stay in a pediatric ward was assumed for the cost per additional child that acquired a nosocomial RSV infection.

UK data on post-hospitalization RSV-associated morbidity indicated that in children with CLD, there was an increase in healthcare resource use attributable to respiratory sequelae for a two-year period after RSV hospitalization [49]. The associated costs were reported at ₤ 14,015 (€ 17,858, EUR 1 = GBP 0.7848, European Central Bank 10 June 2016) per year. Similar cost estimates have not been reported for Spain. Therefore, to account for the increased health care resource use in RSV-H children during their second and third year of life, the UK estimates were rescaled to the Spanish setting. First, by assuming that the RSV-associated respiratory cost is similar in CHD children compared to CLD children, the ratio of the post-hospitalization morbidity costs (i.e. ₤ 14,015) and the hospitalization costs of the first year (i.e. ₤ 19,772 [€ 25,194]) were calculated for the UK (i.e. ratio = 0.71). Second, assuming that the ratio of medical care costs in the second and third year of life versus the first year of life is similar in Spain as in the UK, this ratio was applied to the RSV-hospitalization costs estimated for Spain, eventually yielding Spanish-specific RSV-associated respiratory morbidity costs for the second and third year of life. From the fourth year onwards, yearly costs (both direct and indirect) of respiratory sequelae (asthma and allergic sensitization) were applied in the model for children who developed asthma, sensitization or both [50, 51]. To avoid double-counting of post-hospitalization RSV-associated morbidity costs in RSV-H children, asthma and allergic sensitization costs were not applied in the second and third year of life. In MA-RSV children, asthma and allergic sensitization costs were applied from the second year of life onwards.

### Analyses

Future costs and health outcomes were discounted at 3% in line with the Spanish guideline on economic evaluations of health technologies [52]. The total and incremental life-years (LYs), quality-adjusted life years (QALYs), costs, and the incremental cost-effectiveness ratio (ICER) were estimated. A number of scenario analyses were conducted to test the robustness of the ICER. These scenarios investigated the impact of the following input parameters: model setting parameters (discounting of costs and QALYs, time horizon), clinical parameters (RSV hospitalization probabilities, palivizumab efficacy, case fatality, background mortality, proportion of respiratory sequalae [asthma and/or allergic sensitization], length of respiratory sequelae [asthma and/or allergic sensitization]), utility parameters (baseline utilities, utility decrements [RSV-H and sequelae]) and cost parameters (indirect costs, proportion of palivizumab vials used [50 mg and 100 mg], length of hospital stay of RSV-H patients, cost of nosocomial infections, increase health care resource utilization). A probabilistic sensitivity analysis (PSA) using 3000 simulations was performed to estimate the simultaneous effect of uncertainty surrounding the model parameters. The results of the PSA were used to simulate the joint distribution of the model outcomes. Cost parameters were assumed to follow a gamma distribution whereas utility and risk parameters were assumed to follow a log normal or beta distribution (Table 1 and Table 3).

## Results

Table 4 presents the base case analysis results, both undiscounted and discounted. Fewer patients in the palivizumab arm had MA-RSV infections and RSV-H when compared to the no prophylaxis arm. Considering a hypothetical cohort of 1000 children, palivizumab prophylaxis was estimated to prevent 62 MA-RSV cases and 44 RSV-H cases, including 21 delayed surgeries and four immediate surgeries despite the infection. The prevented MA-RSV and RSV-H cases were predicted to result in fewer patients with long-term respiratory sequelae. In the discounted analysis, over a lifetime horizon, incremental QALYs, LYs, and costs were estimated to be 0.11, 0.07, and € 1693, respectively, yielding an ICER of € 15,748 per QALY gained and € 24,936 per LY gained. The corresponding figures in the undiscounted analysis were 0.21, 0.17, € 1511, € 7212 per QALY gained, and € 9085 per LY gained, respectively.

**Table 4:** Overall survival, quality-adjusted life years and costs per patient, base case analysis

	Undiscounted			Discounted		
	Palivizumab	No prophylaxis	Difference	Palivizumab	No prophylaxis	Difference
Life years	66.51	66.34	0.17	27.15	27.08	0.07
Quality-adjusted life years by RSV history	61.89	61.68	0.21	25.73	25.62	0.11
No RSV	57.61	51.06	6.56	23.96	21.24	2.73
MA-RSV	1.20	5.00	−3.80	0.49	2.07	−1.57
RSV-H	3.08	5.63	−2.55	1.27	2.31	−1.05
Quality-adjusted life years by sequelae history						
No Sequelae	61.28	60.25	1.03	25.25	24.51	0.74
Asthma	0.16	0.34	−0.18	0.13	0.27	−0.14
Allergic sensitization	0.33	0.84	−0.51	0.26	0.66	−0.40
Asthma and allergic sensitization	0.12	0.24	−0.12	0.09	0.19	−0.10
Costs	€ 4731	€ 3220	€ 1511	€ 4574	€ 2881	€ 1693
Prophylaxis costs	€ 3100	€ 0	€ 3100	€ 3100	€ 0	€ 3100
MA-RSV: Medical care	€ 2	€ 9	-€ 7	€ 80	€ 334	-€ 254
MA-RSV: Sequelae	€ 100	€ 418	-€ 318	€ 2	€ 9	-€ 7
RSV-H: Medical care	€ 1063	€ 1941	-€ 879	€ 1042	€ 1904	-€ 862
RSV-H: Sequelae	€ 440	€ 803	-€ 364	€ 326	€ 595	-€ 269
RSV-H: Nosocomial	€ 27	€ 49	-€ 22	€ 27	€ 49	-€ 22

Abbreviations: RSV, respiratory syncytial virus; MA-RSV, medically attended RSV infection; RSV-H, RSV infection resulting in hospitalization

**Fig. 3:**
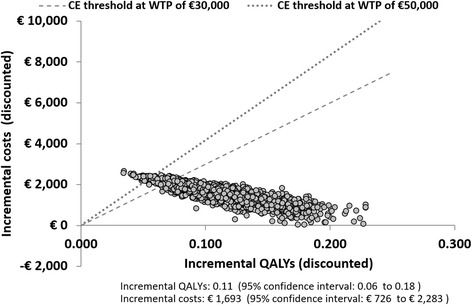
Scatterplot of incremental cost and incremental QALYs. Abbreviations: CE, cost-effectiveness WTP, willingness to pay; QALY, quality-adjusted life year

**Fig. 4:**
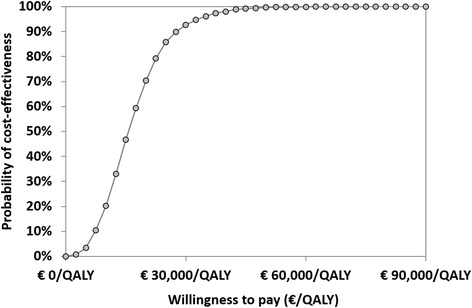
Cost-effectiveness acceptability curve. Abbreviations: QALY, quality-adjusted life year

Probabilistic sensitivity analyses demonstrated that the probabilities of palivizumab prophylaxis being cost-effective at a threshold of € 30,000 per QALY, € 50,000 per QALY and € 100,000 per QALY were 92.7%, 99.6% and 100.0%, respectively. Results of all simulations (100%) fell in the upper right quadrant of the CE plane, denoting both positive incremental QALYs and costs. Simulation results are shown in Fig. 3 (scatter plot of incremental results) and Fig. 4 (cost-effectiveness acceptability curve).

Table 5 presents summary results of the scenario analyses in terms of the ICER per QALY gained. Due to the base case ICER being well below the generally accepted cost-effectiveness threshold, most analyses assessed the detrimental impact of a particular scenario on the ICER. Applying 5% discount rates (costs and effects), considering a 10-year and 30-year time horizon increased the ICER to € 22,009 per QALY, € 33,654 per QALY, and € 19,307 per QALY, respectively. Model results were also sensitive to various assumptions concerning long-term respiratory sequelae. If allergic sensitization was not considered in the model, the ICER increased to € 22,055 per QALY. Expectedly, the ICERs increased further, when both allergic sensitization and asthma were excluded from the evaluation (€ 28,333 per QALY). Additionally, the model was sensitive to the scenario which assumed that in 90% of all prophylaxis administrations a 100 mg vial was used whereas in 10% of all administrations, both, a 50 mg and 100 mg vial was used. This scenario resulted in an increase in the base case ICER to € 26,249 per QALY. Overall, the scenario analyses revealed that the base case ICER was robust to changes in other parameters (e.g. incidence of sequelae, MA-RSV, quality of life). Therefore, it was concluded that palivizumab remained cost-effective even under extreme scenarios.

**Table 5:** Results of the scenario analyses, incremental costs, outcomes, and incremental cost-effectiveness ratios

Scenario	Incremental costs	Incremental QALYs	Incremental LYs	ICER (QALYs)	ICER (LYs)
Base case scenario	€ 1693	0.11	0.07	€ 15,748	€ 24,936
Model setting parameters					
Discount rate: 1.5%	€ 1610	0.14	0.10	€ 11,252	€ 16,005
Discount rate: 5%	€ 1783	0.08	0.05	€ 22,009	€ 38,971
Time horizon: 10 years	€ 1693	0.05	0.02	€ 33,654	€ 77,789
Time Horizon: 30 years	€ 1693	0.09	0.05	€ 19,307	€ 36,405
Clinical parameters					
10% (relative) lower RSV-H rate	€ 1808	0.10	0.06	€ 18,364	€ 29,595
Palivizumab efficacy (RSV-H) at lower 95% confidence interval limit	€ 1916	0.09	0.05	€ 21,300	€ 35,024
Palivizumab efficacy (RSV-H) at upper 95% confidence interval limit	€ 1415	0.13	0.08	€ 10,952	€ 16,805
Case fatality based on Wang et al. (3.72%) [54]	€ 1673	0.09	0.05	€ 18,720	€ 34,629
Case fatality +1% higher in no prophylaxis group	€ 1721	0.13	0.10	€ 12,841	€ 17,825
No general population background mortality in 1st year	€ 1572	0.11	0.07	€ 14,133	€ 23,155
Allergic sensitization excluded	€ 1923	0.09	0.07	€ 22,055	€ 28,328
All respiratory sequelae excluded	€ 2209	0.08	0.07	€ 28,333	€ 32,545
Length of respiratory sequelae 6 years	€ 2020	0.09	0.07	€ 23,512	€ 29,764
Length of respiratory sequelae 12 years	€ 1841	0.10	0.07	€ 18,843	€ 27,125
Length of respiratory sequelae 24 years	€ 1569	0.12	0.07	€ 13,574	€ 23,118
Length of respiratory sequelae: lifetime	€ 1094	0.15	0.07	€ 7446	€ 16,112
Proportion of respiratory sequelae: +5%	€ 1584	0.11	0.07	€ 14,054	€ 23,344
Proportion of respiratory sequelae: −5%	€ 1801	0.10	0.07	€ 17,616	€ 26,529
Utility parameters					
No utility decrement for RSV-H	€ 1693	0.11	0.07	€ 15,947	€ 24,936
No utility decrement for allergy	€ 1693	0.09	0.07	€ 19,414	€ 24,936
No utility decrement for asthma	€ 1693	0.10	0.07	€ 17,224	€ 24,936
Cost parameters					
Indirect costs associated with prophylaxis and hospitalization excluded	€ 1552	0.11	0.07	€ 14,441	€ 22,867
Palivizumab cost: Proportions of 50 mg and 100 mg vial use equal to 0% and 100%, respectively	€ 1751	0.11	0.07	€ 16,295	€ 25,804
Palivizumab cost: Proportion of 100 mg and 50 mg + 100 mg vial use, equals to 90% and 10%, respectively	€ 1930	0.11	0.07	€ 17,954	€ 28,431
Palivizumab cost: Proportion of 100 mg and 50 mg + 100 mg vial use, equals to 40% and 60%, respectively	€ 2821	0.11	0.07	€ 26,249	€ 41,566
Length of stay associated with RSV-H based on the pivotal trial (12.4 days pediatric ward, 38.1% ICU admission, 15.2 days ICU) [16]	€ 1118	0.11	0.07	€ 10,402	€ 16,471
Indirect costs associated with prophylaxis, hospitalization, and respiratory sequelae excluded	€ 1223	0.11	0.07	€ 11,374	€ 18,010
Costs associated with nosocomial infections excluded	€ 1715	0.11	0.07	€ 15,954	€ 25,263
Exclude delayed surgeries pathway from decision tree	€ 1855	0.11	0.07	€ 17,261	€ 27,332
Increased HCU in RSV-H: 0 years	€ 2027	0.11	0.07	€ 18,861	€ 29,866
Increased HCU in RSV-H: 4 years	€ 1860	0.21	0.17	€ 8882	€ 11,189

Abbreviations: QALYs, quality-adjusted life years; LY, life years; ICER, incremental cost-effectiveness ratio; RSV, respiratory syncytial virus; RSV-H, RSV infection resulting in hospitalization; ICU, intensive care unit; HCU, health care resource use

## Discussion

This current study estimated the cost-utility of palivizumab prophylaxis versus placebo in Spain from the societal perspective, using a novel cost-effectiveness model reflecting evidence-based clinical pathways. The base case model results indicated that palivizumab prophylaxis yields additional QALYs and LYs at additional costs. In the discounted analysis, incremental QALYs, LYs, and costs were estimated to be 0.11, 0.07, and € 1693, respectively, yielding an ICER of € 15,748 per QALY gained and € 24,936 per LY gained. The corresponding figures in the undiscounted analysis were 0.21, 0.17, € 1511, € 7212 per QALY gained, and € 9085 per LY gained, respectively. Probabilistic sensitivity analyses demonstrated that the probability of palivizumab prophylaxis being cost-effective at a € 30,000 per QALY threshold was 92.7%.

A recent review of cost-effectiveness studies assessing palivizumab versus no prophylaxis in children with CHD summarized the currently available evidence [53]. This literature review found that only four economic evaluations focused solely on the CHD subpopulation [24–27]. However, several additional analyses examined CHD populations in conjunction with other high-risk subgroups [21, 40, 41, 54–56]. The studies included in the review were fairly recent; most assessments were published in or after 2008 [21, 24, 40, 54–56] and covered a number of countries including Germany [24], the UK [27, 41], and the US [26, 56]. Most studies were based on a cost-utility analysis, i.e., the incremental cost per additional QALY was estimated; however, one evaluation assessed the incremental costs of a prevented hospitalization [25]. In each study, the model structure was based on the design and primary outcome of the pivotal trial [2, 16], so that the reduction of RSV-related hospitalization by palivizumab prophylaxis could be directly reflected. Thus, models typically included RSV hospitalization and non-hospitalization health states. Frequently, the health state capturing RSV hospitalization considered days spent in both ICU and the general pediatric ward. If the cost-effectiveness of palivizumab was assessed in multiple risk groups (e.g. children with CHD and children with CLD), the same model structure was assumed to be applicable to all considered subgroups. None of the studies explicitly incorporated the risk of complicated and delayed surgery due to RSV infection in children with CHD, nor did they consider the risk of MA-RSV infection. Some of the studies took account of the risk of long-term sequelae in children with history of RSV-hospitalization [21, 40, 41, 54, 55]. These studies typically included asthma as the single respiratory sequelae and assumed that it persisted for a limited number of years [21, 40, 41, 54, 55]. While our study adopted the approach used in previous studies that modeled the impact of respiratory sequelae, we used two additional sources [29, 36] to model asthma and allergic sensitization sequelae, linked to MA-RSV. Case fatality rates during RSV hospitalization varied markedly across the studies [21, 24–27, 40, 41, 54–56]. Many studies used a lower case fatality rate for patients who received palivizumab prophylaxis than for those who did not, i.e., in these studies palivizumab prophylaxis was assumed to reduce the risk of case fatality [21, 24, 26, 27, 40, 41, 54–56]. For the current model, equal case fatality rates were applied for children hospitalized for RSV infection.

While the model presented in this paper better reflects clinical pathways compared to past models, there are a number of limitations that need to be considered in the interpretation of the results. One of the limitations is the lack of available recent randomized trial based efficacy data for palivizumab in children with CHD in Spain. In particular, rates of RSV-H utilized in the model were limited to the pivotal RCTs conducted in a multinational setting more than a decade ago. One can speculate that RSV-H rates in children with CHD may have changed over time, perhaps due to improved socioeconomic or other environmental conditions. The impact of such a scenario was assessed in a deterministic analysis (i.e. assuming 10% relative lower RSV-H rate keeping the efficacy of prophylaxis constant); however, the impact of lower RSV-H rate on the ICER was limited and the conclusion of the study did not change. Additionally, decreasing trends of RSV-H rates have not been confirmed for children with CHD in Spain specifically. In contrast, the available evidence from a retrospective study of hospitalizations for RSV bronchiolitis in children aged <1 year, using nationally representative data for Spain, suggest that RSV-H rates did not decrease over the 2004–2012 assessment period [57]. Overall, given the lack of evidence on decreasing RSV-H rates, it is believed that the model used the best available information in this respect. Another limitation of the study was associated with the available evidence on palivizumab drug costs in Spain. A sophisticated drug cost calculation approach was presented in a previous economic evaluation for the UK, where palivizumab costs were estimated by the required dose, i.e., the number of 100-mg or 50-mg vials needed to administer palivizumab each month [17]. The initial dose was calculated using the infants’ average weight at the start of prophylaxis as reported in the pivotal trial (6.1 kg). Infants’ average weight at each subsequent administration was then estimated using UK-specific WHO growth chart data accounting for the preterm birth. The approach predicted that in 60% of the administrations both a 100-mg and a 50-mg vial would be required. While this approach predicted an ICER of € 26,249 per QALY within the present model framework (please see Table 5), based on local expert opinion it was concluded that the baseline average weight and the applied growth rates do not represent the Spanish setting; these would overestimate the required dose and consequently palivizumab drug costs. To adjust for the lower weight of Spanish infants compared to infants from participating countries of the trial (e.g. US, UK, and Germany), an expert opinion-based drug cost calculation approach was employed. The expert indicated that in 5% of all cases of palivizumab administrations a 50-mg vial was used and in 95% of all cases of palivizumab administrations a 100-mg vial was used (i.e. in no case were a 50-mg vial and 100-mg vial used, in combination, for a single administration). There is ample evidence demonstrating that the Spanish population is shorter and hence are likely to weigh less than the US, the UK or German populations [58]. Furthermore, it has been documented that children with CHD experience a decreased growth trajectory compared with their peers [59]; therefore, applying growth rates for drug cost calculations that represent the overall population might not be clinically plausible. Another possible limitation of the present study was the way case fatality and CHD-specific background mortality were applied. Both were applied during the first year of the simulation (i.e. in the decision tree). However, the case fatality was sourced from a study that examined children with CHD hospitalized for severe RSV LRTI [31] and may thus already include mortality due to CHD in addition to mortality due to an RSV infection. Although the same case fatality was used for the prophylaxis arm and placebo arm, there is a possibility of double counting. To assess the potential impact, a scenario analysis was conducted to investigate the effect of implementing CHD-specific background mortality, starting from the second year onwards, so that there was no overlap. Results show that the discounted ICER decreased to € 14,133 per QALY (Table 5), thus, revealing that the approach that was taken in the base case analysis was conservative. Furthermore, another limitation of the model was related to the long-term respiratory sequelae incorporated into the model. In the base case analysis, it was assumed that children who developed asthma or allergic sensitization were affected by these conditions until the age of 18 and not afterwards. While the assumption that respiratory sequelae have a fixed duration was based on previous cost-effectiveness models presented in peer-reviewed publications [29, 54, 55, 60], one may argue that the duration of asthma or allergic sensitization lasts longer (or potentially shorter) than 18 years. Sensitivity analyses were conducted to test the impact of respiratory sequelae on the model results. It was found that the ICER changed moderately in response to various assumptions on the duration of respiratory sequelae. However, even in the extreme scenario when no respiratory sequela were taken into account, the ICER remained below the € 30,000 per QALY threshold.

## Conclusion

In conclusion, the cost-utility analysis using a novel model reflecting evidence-based clinical pathways demonstrated that the high efficacy of palivizumab prophylaxis compared to no prophylaxis resulted in a substantial number of prevented hospitalizations and generated a high number of QALYs gained. Although based on a single trial and associated with uncertainty, there is clear evidence that palivizumab prophylaxis represents a cost-effective treatment option in children with CHD according to generally accepted standards of cost-effectiveness in Spain.

## Abbreviations

AS: Allergic sensitization
CHD: Congenital heart disease
CLD: Chronic lung disease
HRQoL: Health-related quality of life
HUI: Health Utilities Index
ICER: Incremental cost-effectiveness ratio
ICU: Intensive care unit
LRTI: Lower respiratory tract infections
LYs: Life-years
MA-RSV: Medically attended RSV infection
PSA: Probabilistic sensitivity analysis
QALYs: Quality-adjusted life years
RRR: Relative risk reduction
RSV: Respiratory syncytial virus
RSV-H: RSV hospitalization
SE: Standard error
SMR: Standardized mortality ratio
UK: United Kingdom
US: Unites States
WTP: Willingness to pay
